# Supplementary material for: Total Sn-2 Palmitic Triacylglycerols and the Ratio of OPL to OPO in Human Milk Fat Substitute Modulated Bile Acid Metabolism and Intestinal Microbiota Composition in Rats
Source: Nutrients. 2023 Nov 26;15(23):4929. doi: 10.3390/nu15234929 (PMC10708361; doi:10.3390/nu15234929)
Supplement: Supplementary file 1 [file nutrients-15-04929-s001.zip › nutrients-2689762-supplementary.pdf]

Total *Sn*-2 Palmitic Triacylglycerols and the Ratio of OPL to OPO in Human Milk Fat Substitute Modulated Bile Acid Metabolism and Intestinal Microbiota Composition in Rats

Lin Zhu [1], Shuaizhen Fang [1], Hong Zhang [2], Xiangjun Sun [1], Puyu Yang [1], Jianchun Wan [2], Yaqiong Zhang [1*], Weiying Lu [1], Liangli (Lucy) Yu [3]

[1] Institute of Food and Nutraceutical Science, School of Agriculture and Biology, Shanghai Jiao Tong University, Shanghai 200240, China

[2] Wilmar (Shanghai) Biotechnology Research & Development Center Co. Ltd., Shanghai 200137, China

[3] Department of Nutrition and Food Science, University of Maryland, College Park, Maryland 20742, United States

* Corresponding Author:

Yaqiong Zhang, Ph.D. Tel: (86)-21-34204538; Fax: (86)-21-34204538; E-mail: yqzhang2006@sjtu.edu.cn

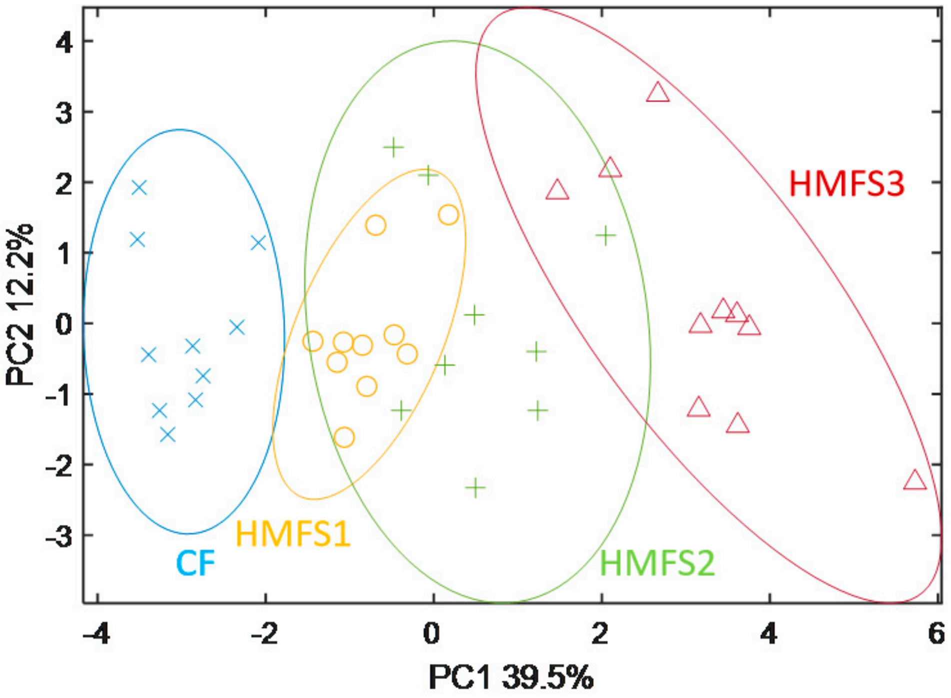

Figure S1. Principal components analysis of hepatic BAs in SD rats.

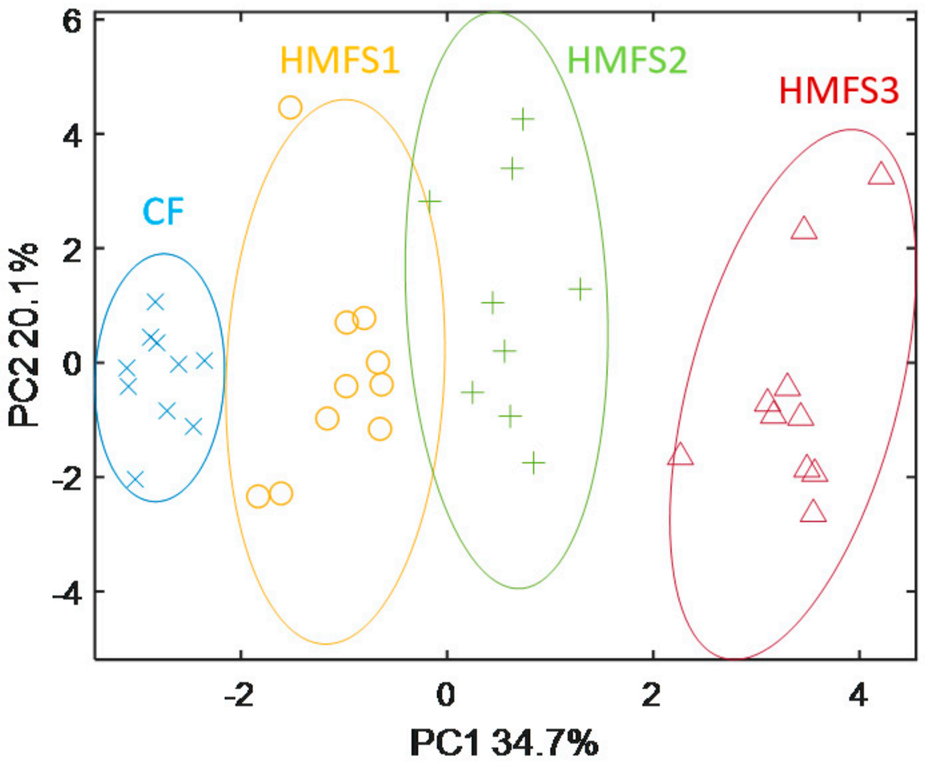

Figure S2. Principal components analysis of ileal BAs in SD rats.

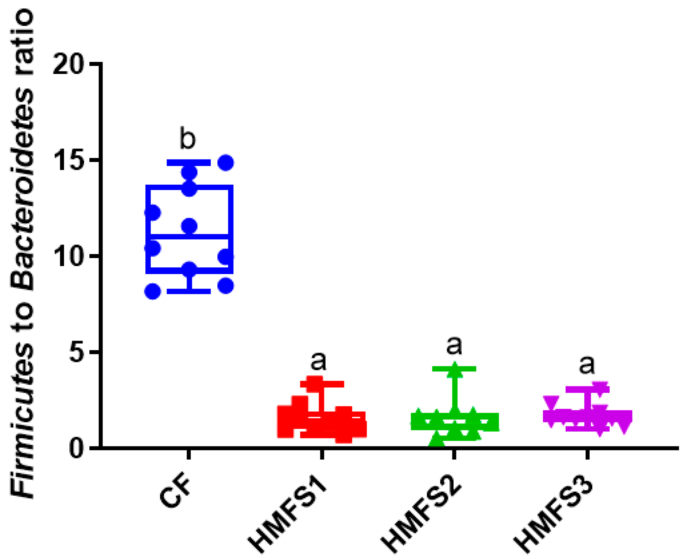

Figure S3. The *Firmicutes* to *Bacteroidete* ratio of gut microbiota in SD rats. Significant differences among the four groups were shown with different letters ($P < 0.05$).

Table S1. Chemical compositions of four experimental fats.

	CF	HMFS1	HMFS2	HMFS3
C8:0 (%)	0.5	0.3	0.4	0.3
C10:0 (%)	0.6	0.6	0.6	0.7
C12:0 (%)	3.5	1.7	2.4	2.1
C14:0 (%)	3.1	3.2	3.2	3.4
C16:0 (%)	21.8	22.1	24.1	23.4
C16:1 (%)	0.3	0.4	0.3	0.4
C18:0 (%)	4.7	4.6	4.7	4.8
C18:1 (%)	41.4	42.1	39.5	39.1
C18:2 n-6 (%)	19.2	20.4	19.8	20.8
C18:3 n-3 (%)	3.0	2.1	2.5	2.6
Sn-2 palmitic acid content (%)	15.5	54.4	60.0	57.9
OPL to OPO ratio	0.4	0.3	0.9	1.4

Table S2. The detailed diet formulations.

	CF		HMFS1		HMFS2		HMFS3	
	gm%	kcal%	gm%	kcal%	gm%	kcal%	gm%	kcal%
Protein	22.2	20.3	22.2	20.3	22.2	20.3	22.2	20.3
Carbohydrate	53.3	48.8	53.3	48.8	53.3	48.8	53.3	48.8
Fat	15.0	30.8	15.0	30.8	15.0	30.8	15.0	30.8
Total		100		100		100		100
Ingredient	gm	kcal	gm	kcal	gm	kcal	gm	kcal
Casein	200	800	200	800	200	800	200	800
L-Cystine	3	12	3	12	3	12	3	12
Corn Starch	255	1020	255	1020	255	1020	255	1020
Maltodextrin 10	132	528	132	528	132	528	132	528
Sucrose	91	364	91	364	91	364	91	364
Celluse, BW200	50	0	50	0	50	0	50	0
Fat Sample CF	137	1233						
Fat Sample HMFS1			137	1233				
Fat Sample HMFS2					137	1233		
Fat Sample HMFS3							137	1233
t-Butylhydroquinone	0.0274	0	0.0274	0	0.0274	0	0.0274	0
Mineral	35	0	35	0	35	0	35	0
Vitamin	10	40	10	40	10	40	10	40
Choline Bitartrate	2.5	0	2.5	0	2.5	0	2.5	0
